# Experimental Science in Modern Education

**Published:** 1875-09

**Authors:** 


Experimental Science in Modern Education.—In the first place, then, I must declare my conviction that no educated man can expect to realize his best possibilities of usefulness without a practical knowledge of the methods of experimental science. If he is to be a physician, his whole success will depend on the skill with which he can use these great tools of modern civilization. If he is to be a lawyer, his advancement will in no small measure be determined by the acuteness with which he can criticise the manner in which the same tools have been used by his own or his opponent's clients. If he is to be a clergyman, he must take sides in the great conflict between theology and science, which is now raging in the world, and, unless he wishes to play the part of the doughty knight Don Quixote, and thinks he is winning great victories by knocking down the imaginary adversaries which his ignorance has set up, he must try the steel of his adversary's blade. Let me be fully understood. It is not to be expected or desired that many of our students should become professional men of science. The places of employment for scientific men are but few, and more in future than in the past they will naturally be secured by those whom Nature has endowed with special aptitudes or tastes—usually the signs of aptitudes—to investigate her laws. That our country will always offer an honorable career to her men of genius, we have every reason to expect, and these born students of Nature will usually follow the plain indications of Providence without encouragement or directions from us. It is different, however, with the great body of earnest students who are conscious of no special aptitudes, but who are desirous of doing the best thing to fit themselves for usefulness in the world ; and I feel that any system of education is radically defective which does not comprise a sufficient training in the methods of experimental science to make the mass of our educated men familiar with this tool of modern civilization : so that when, hereafter, new conquests over matter are announced and great discoveries are proclaimed, they may be able not only to understand but also to criticise the methods by which the assumed have been reached, and thus be in a position to distinguish between the true and the false. Whether we will or not, we must live under the direction of this great power of modern society, and the only question is whether we will be its ignorant slave or its intelligent servant.—PROF. J. P. COOKE, *in Popular Science Monthly for September.*

